# Measurement device-independent quantum key distribution with vector vortex modes under diverse weather conditions

**DOI:** 10.1038/s41598-023-40602-x

**Published:** 2023-09-11

**Authors:** Comfort Sekga, Mhlambululi Mafu

**Affiliations:** 1 Department of Physics and Astronomy, Botswana International University of Science and Technology, P/Bag 16, Palapye, Botswana; 2 Department of Physics, Case Western Reserve University, Cleveland, OH 44106 USA

**Keywords:** Quantum information, Theoretical physics

## Abstract

Most quantum key distribution schemes exploiting orbital angular momentum-carrying optical beams are based on conventional set-ups, opening up the possibility of detector side-channel attacks. These optical beams also suffer from spatial aberrations due to atmospheric turbulence and unfavorable weather conditions. Consequently, we introduce a measurement device-independent quantum key distribution implemented with vector vortex modes. We study the transmission of vector vortex and scalar beams through a turbulent atmospheric link under diverse weather conditions such as rain or haze. We demonstrate that a maximum secure key transmission distance of 178 km can be achieved under clear conditions by utilizing the vector vortex beams, which have been mainly ignored in the literature. When raindrops have a diameter of 6 mm and fog particles have a radius of 0.5 $\upmu$m, the signals can reach 152 km and 160 km, respectively. Since these distances are comparable, this work sheds light into the feasibility of implementing measurement device-independent quantum key distribution using vector vortex modes under diverse weather conditions. Most significantly, this opens the door to practical secure quantum communications.

## Introduction

Quantum key distribution (QKD) allows sharing of information-theoretic cryptographic keys by distant users, even in the presence of a third party with unlimited computational power^1^. Over the past few years, there have been significant advances in the implementation of QKD^2–4^ . Even though QKD has reached this milestone, challenges still need to be overcome before the technology can be fully adopted in real-world applications^5^. Among others, challenges relate to optimal secret key rate-transmission distance limit, infrastructure size and costs, imperfect physical devices, signal-to-noise ratio, and practical security^6–9^. A more practical solution for wide deployment of QKD is chip-based devices which offer advantages such as low cost, low power consumption, well-established batch fabrication techniques, improved performance, miniaturization, and enhanced functionality^10–12^. Other challenges concern imperfections in communication channels, for example, quantum data communications and networking, underwater communication, satellite communication, and fiber-optic communication^1,13–16^. A QKD protocol is ideally secure only when it utilizes perfect single-photon sources and detectors, which are currently unavailable^5,17^. Thus, device imperfections may expose security loopholes or allow side-channel attacks by an eavesdropper, compromising the security of practical implementations. Thus, it is imperative to design protocols robust against device imperfections, such as decoy-state QKD^18,19^ and protocols that are tolerant to reference frame misalignment^20,21^. Another bottleneck to large-scale QKD deployment is high channel loss and decoherence, which results in a relatively low secret key rate^2,16^. Developing efficient methods and models that address these challenges is critical to achieving full-scale practical QKD for secure everyday communications. Therefore, a novel approach, measurement-device-independent QKD (MDI-QKD), was proposed to overcome the communication distance barrier between the participants^22,23^. The scheme allows two users (Alice and Bob) to send their optical signals to an untrusted intermediate node, i.e., Charlie, who performs the measurement, doubling the distance conventional QKD schemes can cover. Most significantly, MDI-QKD removes all detector side-channels from the measurement unit, widely recognized as one of the most vulnerable parts of QKD systems. Remarkably, a variant of the MDI-QKD, named the twin-field QKD^16^, was discovered which is capable of scaling quadratically with channel transmittance marking another milestone towards the realization of long-distance quantum communications. The protocol has been studied in both theory^24–26^ and experimentally^27^ demonstrate its unique advantages.

The MDI-QKD has been extensively studied with optical signals encoded with polarization and phase characteristics of photons^28–35^. Although these degrees of freedom are more suitable for implementations with optical fibers, they are prone to birefringence effects that induce decoherence of signals and require interferometric stability^36^. Therefore, free-space optical links are generally preferred for long-distance QKD communications, especially in areas where fiber installation is not feasible, such as satellite-to-ground links^37–40^. Most significantly, the orbital angular momentum (OAM) states have recently attracted attention in free-space QKD owing to their rotational invariance in the transmission direction, eliminating error rates caused by misalignment of reference frames^41–46^. Furthermore, the OAM theoretically spans infinite Hilbert space, thereby enabling more information to be encoded per photon^47^. Despite this, under bad weather conditions or a turbulent atmosphere, the OAM beam experiences additional broadening, absorption, and backscattering due to random scattering on dust particles, aerosols, and/or precipitation, resulting in the loss of information^48–56^. While there is very limited study on the impact of other weather conditions, such as fog and rain on OAM beams, numerous studies exist regarding quantum optical beam propagation in turbulent atmospheres in the presence of haze and fog^37,57–66^. We highlight that OAM and the vector vortex modes have been recently exploited to analyse the performance of MDI-QKD^67–69^. While these studies are of great importance for QKD, they need more consideration of other practical scenarios that might limit the performance of QKD. For instance, Wang et al.^67^ proposed a MDI-QKD that employs only OAM degree of freedom as carrier of information. However, OAM-carrying beams are more susceptible to losses in the turbulent atmosphere, especially those generated from the superposition of two OAM values. A more promising solution is coupling of polarization and OAM degrees of freedom which has proved to be more resilient to misalignment as they propagate through the turbulent environment. The MDI-QKD employing vortex beams, a hybrid of polarization and OAM degree of freedom, has been introduced in Ref.^68^ . The performance of the protocol was analysed by considering the optical fiber channel. However, transmission of OAM carrying beams via conventional fiber is faced with a challenge of spatial-mode mixing which result in OAM mode information loss. More recently, Li et al.^69^ proposed a similar work on hybrid polarization-OAM MDI-QKD. Their proposed protocol exploits high dimensional vector vortex beams to encode information and further employs a filter-based detection of Bell state set up which utilises six beam-splitters and eight detectors. The use of such detection method would result in lower signal-to-noise ratio due to the losses at the beam-splitters and low detection efficiency attributed to many detectors. In the context of QKD, such a loss would lead to lower yield and effective key rates, jeopardising the advantage provided hybrid polarization-OAM modes. Most significantly, in contrast, our proposed MDI-QKD protocol utilises a simple and easy to implement deterministic method of sorting the vector vortex mode which relies on interference of modes. The method is more efficient to filter based technique in terms of number resources and complexity of the scheme used in Charlie’s measurement site. Notably, this has been demonstrated experimentally to perform better than filter based technique^70^. Another noticeable difference is that in our work, we emulate real-world conditions, particularly by simulating the performance of the protocol under diverse weather conditions. On a daily basis, communication under these weather conditions is inevitable, thus it is vital to evaluate the feasibility of MDI-QKD with vector vortex beams under such conditions. Also, in our protocol we consider the free-space link, which makes our protocol applicable to ground-satellite stations communication. While the work in Ref.^69^ is a significant advance, it only provides the achievable key rates and transmission distances under consideration of optical fiber channel which is susceptible to losses induced by spatial-mode mixing.

Real-world deployment of QKD protocols often entails operating in diverse environments, including turbulent weather conditions. Diverse weather conditions cause interference in communication channels, causing fluctuations in the received signal quality, errors and inadvertently enhancing the potential for eavesdropping. As a result, demonstrating MDI-QKD security under such adverse scenarios provides assurance to withstand the challenges encountered during implementation. This validation is crucial for building trust in QKD systems and encouraging the widespread adoption of secure communication applications. Demonstrating the security of QKD protocols under turbulent weather conditions is a substantial advance toward advancing quantum communication in real-world scenarios. Besides aiding the development of robust QKD systems, this work also lays the foundation for exploring new, efficient, secure quantum communication protocols resilient to adverse weather conditions. Therefore, as a critical aspect of our contribution, we close this gap by proposing an MDI-QKD scheme implemented with vector vortex and scalar beams. This approach maximizes the advantages of both OAM states and MDI-QKD. A specific combination of OAM and polarization modes (hybrid states) provides these optical beams. As a result, any perturbation caused by misalignment of the polarization is precisely compensated for by an identical effect caused by misalignment of the OAM modes. This results in rotationally-invariant photon states. Another novel key aspect of this work is that we examine the performance of the proposed MDI-QKD scheme under diverse weather conditions to determine its viability since communicating information under varied weather conditions is necessary for real-world applications. We do this by evaluating key figures of merit, i.e., secure key rate and transmission distance. These results are of central importance, as they provide valuable insights into the feasibility of MDI-QKD based on vector vortex beams and pave the way for long-distance quantum secure communication.

## Results

### Operation of the OAM based MDI-QKD protocol

We propose an MDI-QKD scheme implemented with vector vortex and scalar beams. Typically, in quantum optics, optical fields can be manipulated to create vector vortex or scalar beams. Vector vortex beams are states of light with spatially varying polarization in the transverse plane, i.e., inhomogeneous polarization states. The spatial and polarization degrees of freedom (DoFs) are coupled in a non-separable manner, reminiscent of entanglement in quantum mechanics. Scalar beams, on the other hand, are completely separable in spatial and polarization modes, i.e., the spatial properties are not affected by changes in the polarization state of the photon. Specifically, vector vortex beams are defined by utilizing the notation adopted from quantum mechanics as^71^:

1 $$\begin{aligned} \vert \psi \rangle _{\theta , \ell }=\frac{1}{\sqrt{2}} (\vert \text {R} \rangle \vert \ell \rangle + e^{i\theta }\vert \text {L}\rangle \vert -\ell \rangle ), \end{aligned}$$

and the mutually unbiased bases (MUB) scalar beams are expressed as

2 $$\begin{aligned} \vert \phi \rangle _{\theta , \ell }= \frac{1}{\sqrt{2}}(\vert \text {R} \rangle + e {^{i\theta }} \vert \text {L}\rangle )\vert \pm \ell \rangle , \end{aligned}$$

where $\text {R}$ and $\text {L}$ correspond to the right and left circular polarization states of light, and $\vert \pm \ell \rangle$ is an OAM state that carries $\pm \ell \hbar$ quanta of OAM. This quantity can be represented as

3 $$\begin{aligned} |\ell \rangle =A(r,z)W(r/R)\exp (i\ell \phi ), \end{aligned}$$

where *A* corresponds to an amplitude of the beam, *r* and $\phi$ are the radial and azimuthal coordinates, respectively. The term $\ell$ denotes an OAM topological charge, and it is an integer, while *W*(*r*/*R*) denotes an aperture function with radius *R* expressed as^50^

4 $$\begin{aligned} W(r/R)= {\left\{ \begin{array}{ll} 1, \ \text {if} \ |r|<R \\ 0, \ \text {otherwise}. \end{array}\right. } \end{aligned}$$

### State preparation

The OAM based MDI-QKD is realized by manipulating the vector vortex and scalar beams in Eqs. (1) and (2) an with intra modal phase $\theta = 0$ or $\pi$ to generate two mutually unbiased bases (MUB), vector basis $\textbf{V} \in \{V_{0}= \frac{1}{\sqrt{2}}(|\text {R}\rangle |\ell \rangle + |\text {L}\rangle |-\ell \rangle ), V_{1}= \frac{1}{\sqrt{2}}(|\text {R}\rangle |\ell \rangle -|\text {L}\rangle |-\ell \rangle )\}$ and the scalar basis $\textbf{S} \in \{S_{0}=\frac{1}{\sqrt{2}}(|\text {R}\rangle +|\text {L}\rangle ) \ |\ell \rangle , S_{1}=\frac{1}{\sqrt{2}}(|\text {R}\rangle -|\text {L}\rangle ) |-\ell \rangle \}$ . The two communication parties, Alice and Bob randomly and independently choose a basis ($\textbf{V}$ or $\textbf{S}$), and a bit $r \in \{0,1\}$ where $r=0 \in \{V_{0},S_{0}\}$ and $r=1 \in \{V_{1},S_{1}\}$.

Next, they generate optical signals of intensity $\gamma \in \{\mu ,\nu ,0\}$ (where $\mu$ is the intensity for signal states, $\nu$ for decoy states, and $\omega$ for vacuum states) prepared in the basis state of $\beta \in \{\textbf{V}, \textbf{S}\}$. Alice and Bob send their states to Charlie via the quantum channel.

**Figure 1:**
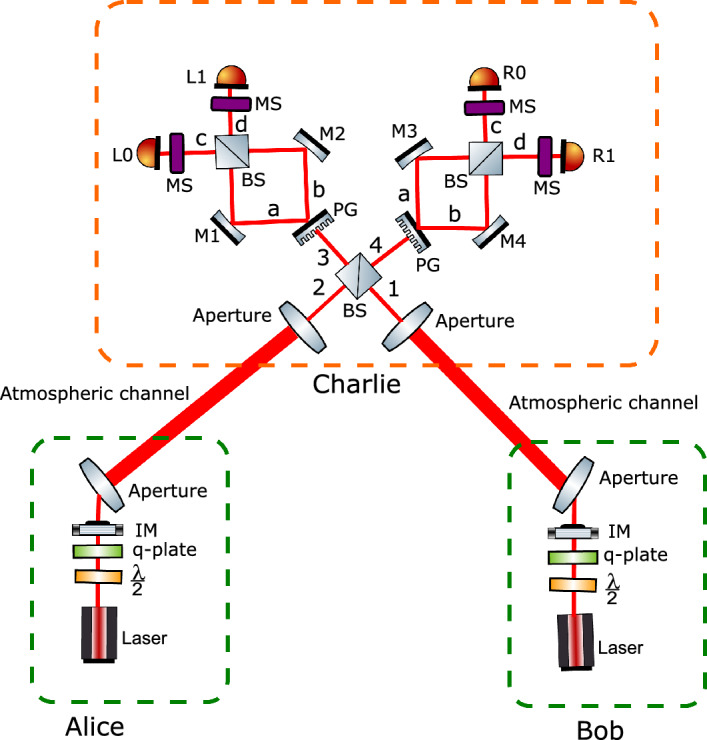
An illustration of the proposed OAM-based MDI-QKD. Alice and Bob prepare two mutually unbiased basis states ($\textbf{V}$, $\textbf{S}$) and send them to Charlie through the unsecure channel. The $\lambda /2$ plate and the *q*-plate are used to generate a set of vector and scalar modes, which are then attenuated to intensity $\gamma \in \{\mu ,\nu ,0\}$ using intensity modulator IM. Next, the telescope collimates the quantum states with a finite aperture. They are then sent through a free atmospheric space link to the measurement site controlled by Charlie. The optical states are then collected by the telescope and allowed to interfere with the symmetric beam splitter (BS). Next, the photons are passed through the polarization grating that separates the left and right circularly polarized photons, then guided by mirrors (M1, M2, M3, M4) towards a beam-splitter (BS). As a result, the photons are then measured using the mode sorters (MS) that map OAM to position and then detected by the detectors (L0, R0, L1, R1). This illustration was generated using Inkscape 1.1 software.

### Measurement

Charlie let the two optical pulses interfere in the symmetric beam splitter (BS) and performs mode sorting and Bell state measurement. When the photons carrying OAM states from Alice and Bob arrive at 50:50 BS, a Hong-Ou-Mandel (HOM) effect occurs. In particular, according to the HOM effect, two indistinguishable photons incident at each input port of BS will exit at the same output port of BS. However, four distinct possibilities exist with distinguishable photons: the two photons exit the BS together through the same output port, or the photons exit the BS separately through different output arms. Precisely, for the initial state $|\Psi \rangle =|1\rangle _{1,\textbf{M}}|1\rangle _{2,\textbf{N}}=|\textbf{M}\rangle _{1} |\textbf{N}\rangle _{2}=\hat{a}^{\dagger }_{1,\textbf{M}}\hat{b}^{\dagger }_{2,\textbf{N}}|0\rangle$, the transformation relations of the two photons incident at the two inputs of BS can be described as

5 $$\begin{aligned} \begin{aligned} {\hat{a}^{\dagger }_{1,\textbf{M}}\hat{b}^{\dagger }_{2,\textbf{N}}|0\rangle \, {\overset{BS}\mapsto}\, }&{\frac{1}{2}(\hat{c}^{\dagger }_{3,\textbf{M}}+\hat{d}^{\dagger }_{4,\textbf{M}})(\hat{c}^{\dagger }_{3, \textbf{N}}-\hat{d}^{\dagger }_{4,\textbf{N}})|0\rangle }\\ {=}&{\frac{1}{2}(\hat{c}^{\dagger }_{3,\textbf{M}}\hat{c}^{\dagger }_{3,\textbf{N}}- \hat{c}^{\dagger }_{3,\textbf{M}}\hat{d}^{\dagger }_{4,\textbf{N}}+ \hat{c}^{\dagger }_{3,\textbf{N}}\hat{d}^{\dagger }_{4,\textbf{M}}-\hat{d}^{\dagger }_{4,\textbf{N}}\hat{d}^{\dagger }_{4,\textbf{N}})|0\rangle , }\end{aligned} \end{aligned}$$

where $\hat{a}^{\dagger }$, $\hat{b}^{\dagger }$, $\hat{c}^{\dagger }$ and $\hat{d}^{\dagger }$ are creation operators at input and output ports 1,2 and 3,4, respectively. The notations $\textbf{M}$ and $\textbf{N}$ correspond to the degree of freedom, such as polarization and orbital angular momentum. For the two input photons with the same degree of freedom that are identical, the second and third terms in Eq. (5) disappears. For simplicity, for initial states, $|\psi \rangle$ and $|\varphi \rangle$ the action of the beam-splitter can also be illustrated as

6 $$\begin{aligned} {|\psi \rangle _{1}|\varphi \rangle _{2}\, {\overset{BS}\mapsto}\, \frac{1}{2}(|\psi \rangle _{3} |\overline{\varphi }\rangle _{3}-|\psi \rangle _{3}|\varphi \rangle _{4}+|\overline{\psi }\rangle _{4} |\overline{\varphi }\rangle _{3}-|\overline{\psi }\rangle _{4}|\varphi \rangle _{4}) }\end{aligned}$$

where $|\bar{x}\rangle$ is the reflected state. Based on the beam-splitter interactions, we describe observations from each basis as follows.

*Vector basis*

Based on a method described in Ref.^71^, two vector vortex states are sorted by combining geometric phase control and multipath interference (see Fig. 1). Once photons exit the first BS, they pass through a polarization grating. This separates left and right circularly polarized photons into two paths based on their polarization according to

7 $$\begin{aligned} \vert \psi \rangle _{\theta , \ell }=\frac{1}{\sqrt{2}}(\vert \text {R} \rangle _{a}\vert \ell \rangle _{a} + e^{i\theta }\vert \text {L}\rangle _{b}\vert -\ell \rangle _{b}). \end{aligned}$$

As a result of interference between the photons in paths *a* and *b* at the second BS, the resultant state is expressed as follows

8 $$\begin{aligned} |\psi 
'\rangle _{\theta ,\ell }=\frac{1-e^{i\theta }}{2}|\ell \rangle _{c}+ {i} \frac{1+e^{i\theta }}{2}|-\ell \rangle _{d}. \end{aligned}$$

Note that due to parity differences in the reflections in each input port, the polarisation of the two paths is inherently reconciled in each output port of the beam splitter. Also, it is worth stating that at this stage, it is not essential to keep the polarisation in the expression of the photon state since the polarisation details are defined in the path. The OAM carrying photons from the outputs *c* and *d* are then measured by passing them through the OAM mode sorter and coincidently detected by two detectors L0 and L1 or R0 and R1. According to Eq. (8) when Alice and Bob prepare the same vector states, we observe a click from one detector along the same path (3 or 4) from the output ports of the first BS. An error corresponds to a click from two detectors within the same path (3 or 4) when the same states are sent by two parties . However, if Alice and Bob prepare vector vortex beams of different states, two detectors are triggered at opposite ends, that is, (L0, R1) or (L1, R0) or within the same path (3 or 4), i.e., (L0, L1) or (R0, R1). For instance, suppose that Alice sent $|\varphi \rangle _{2}=\frac{1}{\sqrt{2}}(|\text {R}\rangle |\ell \rangle - |\text {L}\rangle |-\ell \rangle )$ and Bob sent vector vortex state $|\psi \rangle _{1}=\frac{1}{\sqrt{2}}(|\text {R}\rangle |\ell \rangle + |\text {L}\rangle |-\ell \rangle )$, then based on the first BS interactions we have

9 $$\begin{aligned} \begin{aligned} {|\psi \rangle _{1}|\varphi \rangle _{2}\, {\overset{BS}\mapsto}}\,&{\frac{1}{2}(}[ {\frac{1}{\sqrt{2}}(|\text {R}\rangle |\ell \rangle + |\text {L}\rangle |-\ell \rangle )}] {_{3}\otimes }[ {\frac{1}{\sqrt{2}}(|\text {R}\rangle |\ell \rangle - |\text {L}\rangle |-\ell \rangle )}] {_{3}-}[ {\frac{1}{\sqrt{2}}(|\text {R}\rangle |\ell \rangle + |\text {L}\rangle |-\ell \rangle )}] {_{3}}\\&{\otimes }[ {\frac{1}{\sqrt{2}}(|\text {R}\rangle |\ell \rangle - |\text {L}\rangle |-\ell \rangle )}]_{4}+[ {\frac{1}{\sqrt{2}}(|\text {R}\rangle |\ell \rangle + |\text {L}\rangle |-\ell \rangle )}]_{4}\otimes [\frac{1}{\sqrt{2}}(|\text {R}\rangle |\ell \rangle \\&- |\text {L}\rangle |-\ell \rangle )]_{3}-[ {\frac{1}{\sqrt{2}}(|\text {R}\rangle |\ell \rangle + |\text {L}\rangle |-\ell \rangle )}]_{4}\otimes [\frac{1}{\sqrt{2}}(|\text {R}\rangle |\ell \rangle - |\text {L}\rangle |-\ell \rangle )]_{4}). \end{aligned} \end{aligned}$$

From the above result, we observe that there are four distinct probable scenarios; $25\%$ probability that both $|\psi \rangle _{1}$ and $|\varphi \rangle _{2}$ will exit at output port 3; $25\%$ probability that $|\psi \rangle _{1}$ exit at port 3 and $|\varphi \rangle _{2}$ leaves at port 4; $25\%$ chance that $|\psi \rangle _{1}$ exit at port 4 and $|\varphi \rangle _{2}$ exit at output arm 3; $25\%$ probability that both states leave the BS at output arm 4. Without loss of generality, let us consider a case where $|\psi \rangle _{1}$ exit at port 4 and $|\varphi \rangle _{2}$ exit at port 3, which is indicated by the third term in Eq. (9). After going through a polarization grating, the states transform as follows

10 $$\begin{aligned} {\frac{1}{\sqrt{2}}(|\text {R}\rangle _{3} |\ell \rangle _{3}+e^{i\theta } |\text {L}\rangle _{3}|-\ell \rangle _{3})\, {PG}\, \frac{1}{\sqrt{2}}(|\text {R}\rangle _{3,a} |\ell \rangle _{3,a}+e^{i\theta } |\text {L}\rangle _{3,b}|-\ell \rangle _{3,b}). }\end{aligned}$$

Note that we have introduced a phase factor $e^{i\theta }$, with $\theta = \pi$. After passing through the second BS we obtain

11 $$\begin{aligned} |\psi \rangle _{\theta ,\ell }=\frac{1-e^{i\theta }}{2}|\ell \rangle _{3,c}+i\frac{1+e^{i\theta }}{2}|-\ell \rangle _{3,d}, \end{aligned}$$

Now, substituting back $\theta = \pi$ into Eq. (11) we obtain

12 $$\begin{aligned} |\psi \rangle _{\pi ,\ell }=|\ell \rangle _{3,c}. \end{aligned}$$

For the photon leaving through port 4, we have

13 $$\begin{aligned} \frac{1}{\sqrt{2}}(|\text {R}\rangle _{4} |\ell \rangle _{4}+e^{i\theta } |\text {L}\rangle _{4}|-\ell \rangle _{4}) \, {PG}\, \frac{1}{\sqrt{2}}(|\text {R}\rangle _{4,a} |\ell \rangle _{4,a}+e^{i\theta } |\text {L}\rangle _{4,b}|-\ell \rangle _{4,b}), \end{aligned}$$

where $\theta =0$. After passing through the BS, we obtain

14 $$\begin{aligned} |\psi \rangle _{\theta ,\ell }=\frac{1-e^{i\theta }}{2}|\ell \rangle _{4,c}+i\frac{1+e^{i\theta }}{2}|-\ell \rangle _{4,d}, \end{aligned}$$

Substituting $\theta = 0$ into Eq. (14) we obtain

15 $$\begin{aligned} {|\psi \rangle _{0,\ell }=i|-\ell \rangle _{4,d}.} \end{aligned}$$

Therefore, this scenario will lead to click in detectors L0 and R1. Table 1 depicts the results of other probable occurrences.

*Scalar basis*

Scalar modes are sorted in an analogous manner to vector modes. As a result, the two states that form the basis can be described as follows

16 $$\begin{aligned} \vert \phi \rangle _{\theta , \ell }= \frac{1}{\sqrt{2}} {(} \vert \text {R} \rangle _{a} \vert \ell \rangle _{a} + e {^{i\theta }} \vert \text {L}\rangle _{b} \vert \ell \rangle _{b} {) } \end{aligned}$$

and

17 $$\begin{aligned} \vert \phi \rangle _{\theta , -\ell }= \frac{1}{\sqrt{2}}\vert \text {R} \rangle _{a} \vert -\ell \rangle _{a} + e {^{i\theta }} \vert \text {L}\rangle _{b} \vert -\ell \rangle _{b}. \end{aligned}$$

The modes are separated into two paths *a* and *b* using a polarization grating and then allowed to interfere in the BS. The output state for Eq. (16) is given by

18 $$\begin{aligned} |\psi '\rangle _{\theta ,\ell }=\frac{1-e^{i\theta }}{2}|\text {R}\rangle _{c}|\ell \rangle _{c}+ {i} \frac{1+e^{i\theta }}{2}|\text {L}\rangle _{d}|\ell \rangle _{d}. \end{aligned}$$

and the output state for Eq. (17) is

19 $$\begin{aligned} |\psi '\rangle _{\theta ,-\ell }=\frac{1-e^{i\theta }}{2}|\text {R}\rangle _{c}|-\ell \rangle _{c}+ {i} \frac{1+e^{i\theta }}{2}|\text {L}\rangle _{d}|-\ell \rangle _{d}. \end{aligned}$$

The intramodal phases are chosen to be $\theta =0$ and $\theta =\pi$ for states in Eq. (18) and Eq. (19), respectively. Therefore, the output states can be reduced to $|\psi '\rangle _{\pi ,\ell }=|\text {R}\rangle |\ell \rangle$ and $|\psi '\rangle _{0,-\ell }=i|\text {L}\rangle |-\ell \rangle$. These results indicate that when Alice and Bob prepare the same scalar states, only one detector will be triggered within the relay. Alternatively, if two parties prepare scalar states with opposite OAM, this will result in the click of two detectors in different output paths of the first BS, i.e., a combination of either (L0, R1) or (R0, L1) or the two detectors triggered within the same path from the first BS, that is, (L0, L1) or (R0, R1). There is also an error in this basis if both detectors within the same path (path 3 or 4) are triggered when the same states are sent.

**Table 1:** Probability distribution for Bell state measurement results announced by Charlie when both Alice and Bob choose the same basis.

Basis	Alice	Bob	Charlie’s measurement results			
			$|\Psi \rangle ^{+}$		$|\Psi \rangle ^{-}$	
			L0, L1	R0, R1	L0, R1	L1, R0
Vector basis	$V_{0}$	$V_{0}$	0	0	0	0
	$V_{0}$	$V_{1}$	0.25	0.25	0.25	0.25
	$V_{1}$	$V_{0}$	0.25	0.25	0.25	0.25
	$V_{1}$	$V_{1}$	0	0	0	0
Scalar basis	$S_{0}$	$S_{0}$	0	0	0	0
	$S_{0}$	$S_{1}$	0.25	0.25	0.25	0.25
	$S_{1}$	$S_{0}$	0.25	0.25	0.25	0.25
	$S_{1}$	$S_{1}$	0	0	0	0

### Announcement

Following photon detection, Charlie announces successful measurement events. A successful detection event corresponds to a coincidence click in two detectors (associated with orthogonal OAM). In the proposed protocol, the detectors L0 and R0 are used to detect OAM state $|\ell \rangle$ while L1 and R1 are used to detect OAM state $|-\ell \rangle$. Thus, a combination of (L0, L1), (R0, R1) , (L0, R1) and (R0, L1) corresponds to successful detection. We define a click in detectors (L0,L1) or (R0, R1) to indicate projection into Bell state $|\Psi \rangle ^{+}=\frac{1}{\sqrt{2}}(|01\rangle _{\text {LL}}+|10\rangle _{\text {RR}})$, while a click in detectors (L0, R1) or (R0, L1) correspond to Bell state $|\Psi \rangle ^{-}=\frac{1}{\sqrt{2}}(|01\rangle _{\text {LR}}-|10\rangle _{\text {LR}})$.

### Sifting

When Charlie announces a successful Bell state measurement result, Alice and Bob publish their basis choices and intensity over an authenticated public channel. Bob flips his key bits to match Alice’s as illustrated in Table 2.

**Table 2:** Post-processing of raw key in the sifting step. Bob flips his bits to ensure correct correlation with Alice’s bit.

Alice and Bob	Charlie’s measurement results	
	$|\Psi \rangle ^{+}$	$|\Psi \rangle ^{-}$
Vector basis	Bit flip	Bit flip
Scalar basis	Bit flip	Bit flip

The random bit values $r \in \{0,1\}$ in each basis are assigned as $r=0 \in \{V_{0},S_{0}\}$ and $r=1 \in \{V_{1},S_{1}\}$. The random bits obtained from the vector basis are then exploited by Alice and Bob in order to form a raw key. The random bits from the scalar basis are used to estimate the upper bound in eavesdropper’s information. Then, the two communicating parties perform error correction and privacy amplification in order to obtain a secret key that can be used for secure communication.

### Security analysis

We provide a security analysis for our scheme along the lines of Ref^29^, which makes use of a photon-number channel model and the Gottesman-Lo-Lütkenhaus-Preskill (GLLP) security proof^72^. In particular, the security proof is based on time-reversed EPR-based QKD protocol and the notion of virtual protocol. In this virtual setting, it is assumed that Alice possesses a virtual qubit, and she entangles it with a quantum signal she prepared before sending it to Charlie. Similarly, Bob uses a virtual qubit to prepare an entangled state with the quantum signal he sends to Charlie. Now, in principle, the two could rather store their virtual qubits in her quantum memory and wait for the announcement of successful Bell state measurements by Charlie. The successful measurements of the signals sent by Alice and Bob automatically imply that their virtual qubits are entangled by virtue of entanglement swapping. After that, Alice and Bob can now perform a measurement on their virtual qubits to determine which state they are sending to Charlie. In such virtual qubits setting, the protocol is directly equivalent to an entanglement-based protocol and its security can be proved following the technique proposed in Ref^73^. The key rate formula for the proposed MDI-QKD is given by

20 $$\begin{aligned} K \ge q \{Q_{\mu _{a}\mu _{b}}^{\textbf{V}}f_{\textrm{EC}}H(E_{\mu _{a}\mu _{b}}^{\textbf{V}}) +Q_{11}^{\textbf{V}}(1-H(e_{11}^{\textbf{S}}))\}, \end{aligned}$$

where *q* is the basis sift factor; parameter $\mu$ denotes the signal intensity; $Q_{\mu _{a}\mu _{b}}^{\textbf{V}}$ and $E_{\mu _{a}\mu _{b}}^{\textbf{V}}$ are the overall gain and quantum bit error rate (QBER) in the vector basis, respectively. The quantities $Q_{11}^{\textbf{V}}$ and $e_{11}^{\textbf{S}}$ indicate the gain and error rate of individual photon components. We evaluate these parameters using the decoy state theory presented in the “Methods” section.

### Propagation through perturbing media

We examine how the OAM-carrying optical beams employed in our proposed protocol are affected by various weather conditions during their propagation through the free space link. When OAM beams propagate through atmospheric channels, they undergo aberrations, primarily caused by beam extinction and turbulence effects. Extinction occurs due to absorption and scattering by molecules and aerosols, as opposed to the latter caused by changes in the refractive index of the atmosphere. Atmospheric effects are strongly related to the transmittance, $\eta$, which is defined as the probability of a photon being successfully transmitted through the channel and being detected. This is a critical factor in evaluating QKD protocol performance. To study the influence of various weather conditions on the transmission of OAM signals in the MDI-QKD protocol, we make use of some well-developed atmospheric optical communications models.

### OAM carrying photons through turbulence

As OAM states propagate through free space, their purity is compromised due to the turbulence that occurs in the atmosphere. As a result of fluctuations in the refractive index of the atmosphere caused by turbulence, a propagating optical beam will exhibit random phase aberrations. Based on the methods described in Ref.^50^, we investigate the effects of random phase aberrations on the received OAM state. First, the original optical field at the transmitter is assumed to be given by

21 $$\begin{aligned} A(\textbf{r})=A_{0}W(r/R)e^{i\ell \phi }, \end{aligned}$$

where $A_{0}$ is the (spatially uniform) field amplitude, and other parameters are defined as in Eq. (3). After undergoing scrambling in the turbulent atmosphere, the field at the receiver aperture can be represented as

22 $$\begin{aligned} V(\textbf{r})=A_{0}W(r/R)e^{i\ell \phi }e^{i \vartheta (\textbf{r})}, \end{aligned}$$

where $\vartheta (\textbf{r})$ represents the turbulence-induced wavefront distortion at the receiver. Notably, the quantity $\exp (i\vartheta (\textbf{r}))$ can be expanded in the Fourier series as

23 $$\begin{aligned} e^{i\vartheta (r,\phi )}=\sum _{\ell =-\infty }^{\infty }C_{k}(r)e^{ik \phi }, \end{aligned}$$

where the expansion coefficients $C_{k}(r)$ are given by

24 $$\begin{aligned} C_{k}(r)=\frac{1}{2\pi }\int _{0}^{2\pi }\textrm{d}^{i\vartheta (r,\phi )}e^{-ik \phi }. \end{aligned}$$

The received field $V(\textbf{r})$ can be expanded in a similar manner as $V(r,\phi )=\sum _{\ell =-\infty }^{\infty }V_{n}(r)\exp (in\phi )$, where each Fourier component $V_{n}(r)$ is given by

25 $$\begin{aligned} V_{n}(r)=\frac{1}{2\pi }\int _{0}^{2\pi }\textrm{d}\phi V(r,\phi ) e^{-in \phi }. \end{aligned}$$

By substituting Eqs. (22) and (23) into Eq. (25) yields

26 $$\begin{aligned} V_{n}(r)=\frac{A_{0}}{2\pi }W(r/R)\sum _{\ell =-\infty }^{\infty } C_{\ell }(r)\int _{0}^{2\pi }\textrm{d}\phi e^{-i(n-\ell -m) \phi }. \end{aligned}$$

The above expression can be further reduced to

27 $$\begin{aligned} V_{n}(r)=\frac{A_{0}}{2\pi }W(r/R)C_{\Delta }(r), \end{aligned}$$

by considering that the integral in Eq. (25) equals $2\pi$ when $m-k-\ell =0$ and vanishes otherwise. The last expression defines $\Delta$ as $\Delta =m-\ell$. The connection between azimuthal Fourier components $C_{\Delta }(r)$ associated with atmospheric turbulence and the OAM state of the received field emanating from Eq. (27) allows one to determine the amount of radiation that remains in the initial OAM state based on the spatial component of the azimuthal Fourier spectrum $\exp (i\vartheta (\textbf{r}))$. Practically, this radiation is determined in terms of power contained in each OAM state of the received field. The total power collected by the receiver is given by

28 $$\begin{aligned} P=\frac{1}{2}\epsilon _{0}\int \textrm{d}\textbf{r}W(r/R) V^*(\textbf{r})V(\textbf{r})=\frac{1}{2}\epsilon _{0}|A_{0}|^{2}\pi R^{2}, \end{aligned}$$

Accordingly, this power is constituted by a combination of different (orthogonal) modes of OAM modes of the field according to

29 $$\begin{aligned} P=\sum _{\Delta =-\infty }^{\infty }P_{\Delta }, \ \textrm{where} \ P_{\Delta }= 2\pi |A_{0}|^{2}\int _{0}^{R}\textrm{d}rrC_{\Delta }^{*}(r)C_{\Delta }(r). \end{aligned}$$

An important parameter of interest is the ratio $\eta _{\textrm{turb}}=P_{\Delta }/P$ of the power contained in each OAM mode given by

30 $$\begin{aligned} \eta _{\textrm{turb}}=\frac{2}{R^{2}}\int _{0}^{R}\textrm{d}rrC_{\Delta }^{*}(r)C_{\Delta }(r). \end{aligned}$$

Using this parameter, we can determine the probability that the OAM quantum number *m* of the received state differs from that of the transmitted state $\ell$ by the amount $\Delta =m-\ell$. The result presented in Eq. (30) applies to any realization of atmospheric turbulence. Generally, $\eta _{\textrm{turb}}$ is described as an ensemble average according to the form

31 $$\begin{aligned} \eta _{\textrm{turb}}&= K\int _{0}^{R}\textrm{d}rr \int _{0}^{2\pi } \textrm{d}\phi _{1} \int _{0}^{2\pi } \textrm{d}\phi _{2}\langle e^{-[\vartheta (r,\phi _{1})-\vartheta (r,\phi _{2})]}\rangle \nonumber \\&\times e^{i\Delta (\phi _{1}-\phi _{2})}, \end{aligned}$$

where $K=1/(2\pi ^{2}R^{2})$. By considering the Kolmogorov turbulence theory, the above expression can be further reduced to

32 $$\begin{aligned} \eta _{\textrm{turb}}&= \frac{1}{\pi }\int _{0}^{1}\textrm{d}\rho \rho \int _{0}^{2\pi }\textrm{d}\phi e^{-3.44(D/r_{0})^{5/3}(\rho \sin (\phi /2))^{5/3}}\nonumber \\&\times \cos (\Delta \phi ), \end{aligned}$$

where $\rho =r/R$. Here *D* denotes the receiver aperture diameter, and the parameter $r_0$ corresponds to Fried’s coherence diameter, which is described as

33 $$\begin{aligned} r_0=0.1853\Bigg (\frac{\lambda ^{2}}{C^{2}_{n}L}\Bigg ), \end{aligned}$$

where $\lambda$ is the wavelength of the optical beam, *L* is the transmission distance and $C^{2}_{n}$ is the refractive-index structure parameter, which gives the strength of atmospheric turbulence.

### OAM carrying photons through rain

OAM carrying beams are also highly susceptible to adverse weather conditions, such as rain. Generally, rain attenuates beam energy in free space link QKD due to the absorption and scattering of rain droplets. The phenomenon is known as rain extinction^62^. An empirical formula has been developed to measure rain extinction in relation to rainfall intensity, and is defined as^62,74^:

34 $$\begin{aligned} \alpha _{\textrm{rain}}=1.45 I_{\textrm{rain}}^{0.64}, \end{aligned}$$

where $I_{\textrm{rain}}$ is the rainfall intensity, and $\alpha _{\textrm{rain}}$ is the rain extinction. Using Law-Parsons raindrop size distribution, rainfall intensity is also related to raindrop size as^59,62^:

35 $$\begin{aligned} I_{\textrm{rain}}=6\pi \times 10^{-4} \int _{0}^{\infty }n(D_{\text {rain}}) D_{\text {rain}}^{3}v(D_{\text {rain}})dD_{\text {rain}}. \end{aligned}$$

The above expression can be further simplified by dropping the integral and expressed analytically as

36 $$\begin{aligned} I_{\textrm{rain}}=\frac{6\pi n(D_{\text {rain}})D_{\text {rain}}^{3}v(D_{\text {rain}})}{10^{4}m(D_{\text {rain}})}, \end{aligned}$$

where $D_{\text {rain}}$ denotes diameter of the rain droplet, $n(D_{\text {rain}})$ corresponds to the number of rain-droplets, $v(D_{\text {rain}})$ is terminal velocity of rain-droplets and $m(D_{\text {rain}})$ is percentage of volume.

The transmittance associated with rain extinction for horizontal paths with length *L* is given by

37 $$\begin{aligned} \eta =e^{-\alpha _{\textrm{rain}} L }. \end{aligned}$$

### OAM carrying photons through a foggy atmosphere

This section examines the effects of foggy weather conditions on free space QKD. In general, fog is composed of a large number of small water droplets suspended in the air. Beam degradation caused by fog particles is largely reflected in scattering and absorption contributions, which is known as beam extinction. There are two main factors that influence the extinction effects: the radius of the fog particle and the wavelength of the beam. For modeling the scattering and absorption effect, we consider the Mie scattering theory^75^, which is more appropriate for evaluating the scattering of particles approximately the wavelength of a beam of light. The Mie theory uses Maxwell equations to characterize beam extinction induced by fog particles. We consider the case of a beam perturbed by homogeneous spherical particles that are isotropic. According to Ref.^76^, the relationship between scattered and incident beams is defined as a function of the amplitudes of electric field components as

38 $$\begin{aligned} \begin{aligned} \begin{bmatrix}E^{V}_{S}\\ E_{S}^{H}\end{bmatrix}&=\frac{\exp (ikr)}{-ikr}\begin{bmatrix} S_{1} &{} S_{3} \\ S_{2} &{} S_{4} \end{bmatrix} \begin{bmatrix}E_{V}^{i}\\ E_{H}^{i}\end{bmatrix}. \end{aligned} \end{aligned}$$

The subscripts *V* and *H* refer to the vertical and polarization components of the electric field, respectively. The parameter *k* represents the wave number, and the element $S_{i}$ represents the scattering matrix. Its value is determined by particle diameter, refractive index, beam wavelength, and scattering polar angle. According to the scattering matrix, the value is determined by the particle shape, scale, and refractive index. In the case of spherical particles, $S_{3}=0$, $S_{4}=0$, and the complex solution of the other elements, $S_{1}$ and $S_{2}$, can be written as follows

39 $$\begin{aligned} S_{1}(\theta )&=\sum _{n=1}^{\infty }\frac{2n+1}{n(n+1)}(a_{n}\pi _{n}+b_{n}\tau _{n}),\nonumber \\ S_{2}(\theta )&=\sum _{n=1}^{\infty }\frac{2n+1}{n(n+1)}(b_{n}\pi _{n}+a_{n}\tau _{n}), \end{aligned}$$

where

40 $$\begin{aligned} \pi _{n}&=\frac{P_{n}^{1}(\cos \theta )}{\sin \theta }=\frac{dP_{n}(\cos \theta )}{d(\cos \theta )} \end{aligned}$$

41 $$\begin{aligned} \tau _{n}&=\frac{dP_{n}^{1}(\cos \theta )}{d(\cos \theta )}. \end{aligned}$$

According to the above expressions, $P_{n}^{1}(\cos \theta )$ is the first kind of Legendre function of order *n*. The scattering polar angle is defined by the parameter $\theta$. The Mie scattering quantities $a_{n}$ and $b_{n}$ defined in Eq. (39) are obtained from the Bessel functions as

42 $$\begin{aligned} a_{n}&= \frac{\psi _{n}(x)\psi _{n}'(mx)-m\psi _{n}'(x)\psi _{n}(mx)}{\xi _{n}(x)\xi _{n}'(mx)-m\xi _{n}'(x)\xi _{n}(mx)}, \end{aligned}$$

43 $$\begin{aligned} b_{n}&= \frac{m\psi _{n}(x)\psi _{n}'(mx)-\psi _{n}'(x)\psi _{n}(mx)}{m\xi _{n}(x)\xi _{n}'(mx)-\xi _{n}'(x)\xi _{n}(mx)}, \end{aligned}$$

where for some variables *y*, $\psi _{n}(y)=\sqrt{\frac{\pi y}{2}}J_{n+1/2}(y)$, $\xi _{n}(y)=\sqrt{\frac{\pi y}{2}}H_{n+1/2}(y)$ and $J_{n+1/2}(X)$, $H_{n+1/2}(X)$ denote the first and second kind of semi-integral order Bessel function and Hankel function, respectively. The parameter *m* represents the refractive index of fog particles, which is estimated as $m=1.33+i0.003$, while the quantity *x* is related to the particle’s circumference and wavelength $\lambda$ according to

44 $$\begin{aligned} x=\frac{2\pi R_{\text {fog}}}{\lambda }, \end{aligned}$$

where $R_{\text {fog}}$ is the particle’s radius.

The coefficients $a_{n}$ and $b_{n}$ are useful for determining the extinction efficiency factor caused by fog particles, which can be obtained by^75^

45 $$\begin{aligned} Q_{\text {ext}}=\frac{\lambda ^{2}}{2\pi }\sum _{n=1}^{\infty }(2n+1)\text {Re}(a_{n}+b_{n}). \end{aligned}$$

Thus, the beam attenuation coefficient of fog can be calculated as follows

46 $$\begin{aligned} \alpha _{\text {fog}}=\pi R_{\text {fog}}^{2}Q_{\text {ext}}N_{\text {fog}}, \end{aligned}$$

where $N_{\text {fog}}$ denotes the number of particles per unit volume.

### Simulation

Based on the simulation parameters provided in Table 3, we analyze the performance of OAM-based MDI-QKD under various weather conditions. To begin with, we examine what impact turbulent atmospheric conditions have on the transmitted OAM states. We evaluate the probabilities of obtaining different OAM measurements, $\eta _{\textrm{turb}}$ for OAM beams propagating under Kolmorogov turbulence using Eq. (32).

**Figure 2:**
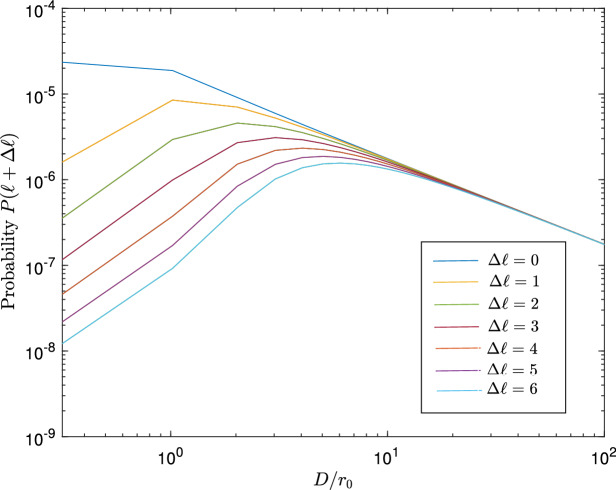
This plot illustrates the probability of receiving adjacent OAM states (Transmittance), $\eta _{\text {turb}}$ against the ratio of the aperture diameter *D* to the Fried parameter $r_{0}$.

**Figure 3:**
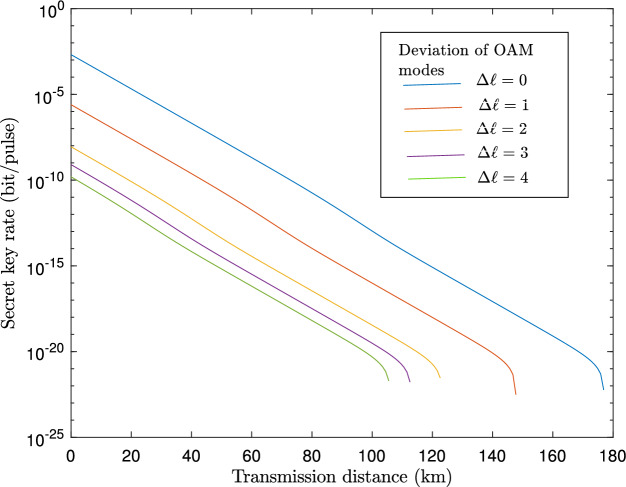
A plot of the secret key generation rate, *K*, versus transmission distance when atmospheric turbulence is varied as measured by the deviation of OAM modes, $\Delta \ell$.

According to Fig. 2, as the receiver aperture diameter *D* becomes comparable to the Fried parameter $r_0$, the probability of obtaining the original OAM states $(\Delta \ell =0)$ at the receiver aperture decreases asymptomatically. When $D\le r_0$ is small, phase aberrations are weak, and OAM scattering is small, but as the Fried parameter approaches the receiver aperture diameter, it becomes more likely that OAM scattering will occur. The curves showing the probability of receiving scrambled OAM states i.e., $\Delta \ell >0$ initially increase with increasing turbulence levels and ultimately decrease with further increase. At high turbulence levels, optical power is spread across various OAM modes, resulting in a decreased probability of detecting a specific OAM value.

Figure 3 shows a relationship between key rate and transmission distance for different deviations $\Delta \ell$ from original OAM states induced by turbulence. The results demonstrate that the key rate and maximum transmission distance decrease with increasing deviation from originally transmitted OAM states. Notably, we observe that the achievable key rate remains comparable to the normal condition without deviation of transmitted OAM states for a lower aberration of OAM states, e.g., for $\Delta \ell =1$.

**Table 3:** Parameters used for simulation.

Parameters						Values
Background count rate						$8 \times 10^{-6}$
Error correction efficiency *f*						1.15
Detector efficiency						14.5%
Aperture diameter, *D*						$15 \times 10^{-2}$ m
$C_{n}^{2}$						$10^{-14} \text {m}^{-2/3}$
Wavelength of the beam, $\lambda$						1550 nm
Terminal velocity of rain, $v\,(D_{\text {rain}})$						9 m/s
Rain-drop size distribution , $n\,(D_{\text {rain}})$						$10^{5}$
Percentage rain volume, $m\,(D_{\text {rain}})$						1
Number of particles per unit volume, $N_{\text {fog}}$						$10^{15}$

**Figure 4:**
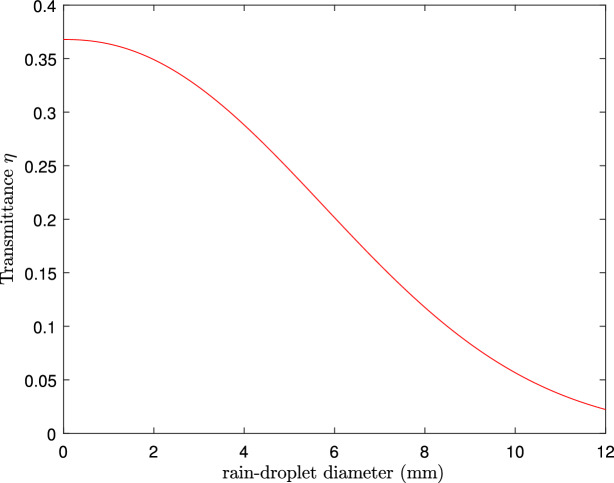
A plot of transmittance, $\eta$, against the diameter of raindrops, $D_{\text {rain}}$.

**Figure 5:**
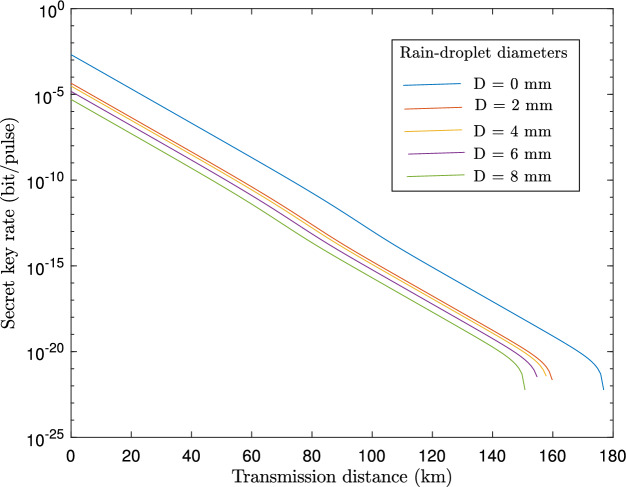
Plot of the secret key generation rate, *K*, against transmission distance in km for a range of raindrop diameters.

**Figure 6:**
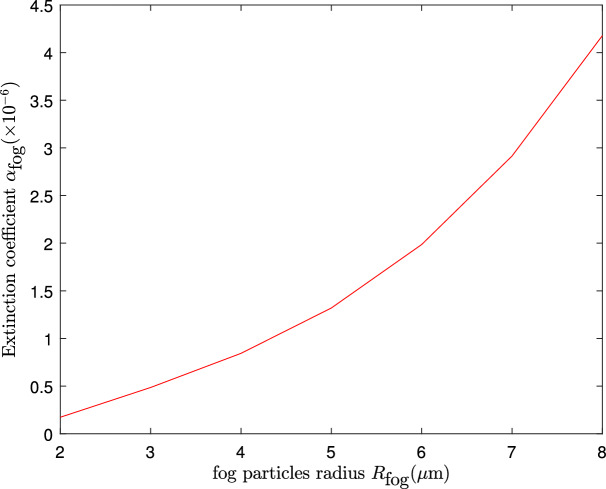
An illustration showing the relation between the beam extinction coefficient, $\alpha _{\text {fog}}$, and the radius of the fog particles, $R_{\text {fog}}$.

**Figure 7:**
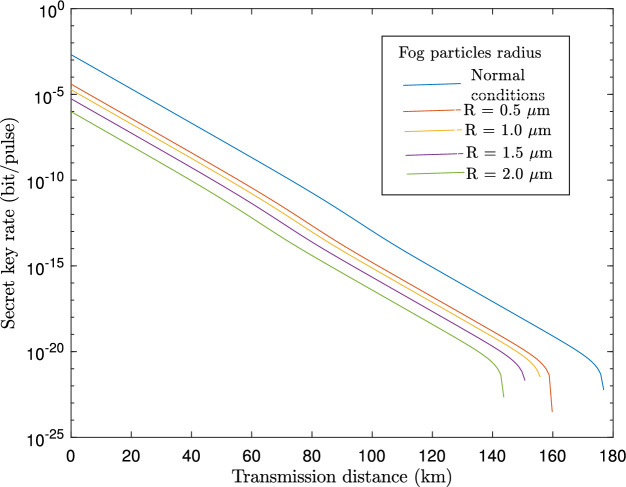
A plot of the secret key generation rate, *K*, against transmission distance in km for various values of the radius of the fog particles.

In Fig. 4, we analyze the impact of rain droplet size on transmittance based on the Law-Parson model depicted in Eq. (36). The transmittance decreases as the size of the rain droplets increases. As the size of rain droplets increases from 3 mm onwards, a sharp drop in transmittance is observed. In Fig. 5, we plotted the achievable key rate against the transmission distance for various sizes of rain droplets. As can be seen from the results, larger rain droplets (which are directly proportional to rainfall intensity) negatively influence key rate and transmission distance. Also, we observe that with a clear atmosphere, the maximum transmission distance is approximately 178 km, while with a rainfall of 2 mm diameter droplets, the maximum distance is 160 km. It should be noted that the distance is further reduced with an increase in the diameter of the rain droplets. A further study was carried out to evaluate the influence of fog particle size on the extinction of optical signals in Fig. 6 based on Eq. (46). Based on the results, the extinction coefficient increases as the fog particle radius increases, indicating an increase in optical absorption and scattering. In Fig. 7, we generated curves for key rates against transmission distances for different sizes of fog particles. Clearly, the achievable key rate and maximum transmission distance decrease as the fog particle size increases. However, we discover that the protocol’s performance under foggy conditions is still comparable to the performance under typical atmospheric conditions. For instance, if we set the real-life parameters for the key rate, $R=10^{-10}$, then the maximum transmission distance under fog particles of size $1\, \upmu$m is 80 km, and under normal conditions, the maximum attainable transmission distance is 100 km.

## Discussion

We have demonstrated free space MDI-QKD using vector vortex and scalar beams. Due to the rotational invariance property of the beams, two communicating parties can generate secret keys without having to align the reference frames of the transmitting and receiving units. Additionally, we evaluated the performance of the proposed protocol under a variety of weather conditions that approximate the realistic conditions of everyday communications. We observed that propagation of OAM carrying beam under turbulent conditions may result in scrambling of the OAM state of the beam, and the probability of scrambling increases as the strength of the turbulence increases. Results indicate that large deviations in originally transmitted OAM states of the vortex and scalar beams lead to reduced achievable key rates and maximum transmission distances. In particular, in a weak turbulence regime, i.e., with a small $\Delta \ell$, the achievable distance is comparable to that under normal atmospheric conditions. Notably, we have also demonstrated that, under clear atmospheric conditions, our proposed scheme can transmit signals up to 178 km. In constrast, with rainfall of 6 mm diameter droplets, the distance to which the signals can be transmitted is 152 km. It should be noted that in foggy conditions with fog particles with a radius of 0.5 $\upmu$m, the maximum attainable distance is 160 km, which is still comparable to the maximum distance reached under clear conditions. These results demonstrate the robustness of MDI-QKD implementation using vector vortex and scalar beams to generate secure keys over long transmission distances in adverse weather conditions. As a result, this study is of central importance as it opens up the intriguing possibility of utilizing these beams in future QKD applications.

## Methods

This section presents the derivation of the parameters used to estimate the MDI-QKD secret key rate. The gain for single photon states $Q_{11}^{\textbf{V}}$, which represents the probability of Alice and Bob sending out single photon states on a vector basis and obtaining successful detection results, is expressed as follows

47 $$\begin{aligned} Q_{11}^{\textbf{V}}=\mu _{a}\mu _{b}e^{-\mu _{a}-\mu _{b}}Y_{11}^{\textbf{V}}. \end{aligned}$$

The quantity $Y_{11}^{\textbf{V}}$ corresponds to the yield of single photons in the vector basis and is given by

48 $$\begin{aligned} Y_{11}^{\textbf{V}}=Y_{11}^{\textrm{L0R1}}+Y_{11}^{\textrm{L1R0}} +Y_{11}^{\textrm{L0L1}}+Y_{11}^{\textrm{R0R1}}+Y_{11}^{\textrm{L0R0}}+Y_{11}^{\textrm{L1R1}}. \end{aligned}$$

Without a loss of generality, here we show how to obtain $Y_{11}^{\textrm{L0R1}}$, and owing to symmetry, other terms are deduced similarly. After propagating through a lossy channel modeled by transmittance $\eta _{a}$, $\eta _{b}$, the initial state of Alice and Bob can be described as a mixed state

49 $$\begin{aligned} \frac{\eta _{a}\eta _{b}}{4}|\psi _{11}\rangle \langle \psi _{11}|+\frac{\eta _{a}(1-\eta _{b})}{2}|\psi _{10}\rangle \langle \psi _{10}|+\frac{(1-\eta _{a})\eta _{b}}{2}|\psi _{01}\rangle \langle \psi _{01}|+(1-\eta _{a})(1-\eta _{b})|\psi _{00}\rangle \langle \psi _{00}| \end{aligned}$$

where $|\psi _{00}\rangle =|{00}\rangle _{ab}$, $|\psi _{01}\rangle =|01\rangle _{ab}$, $|\psi _{10}\rangle =|{10}\rangle _{ab}$, $|\psi _{11}\rangle =|11\rangle _{ab}$ and $|{0}\rangle$, $|{1}\rangle$ represent vacuum and one photon states. After passing through a BS, the states in Eq. (49) transform to

50 $$\begin{aligned} |{11}\rangle _{12}&\longmapsto \frac{1}{\sqrt{2}}(|{0}\rangle _{3}|{2}\rangle _{4}-|{2}\rangle _{3}|{0}\rangle _{4}),\nonumber \\ |{10}\rangle _{12}&\longmapsto \frac{1}{\sqrt{2}}(|{0}\rangle _{3}|{1}\rangle _{4}-|{1}\rangle _{3}|{0}\rangle _{4}),\nonumber \\ |{01}\rangle _{12}&\longmapsto \frac{1}{\sqrt{2}}(|{1}\rangle _{3}|{0}\rangle _{4}-|{0}\rangle _{3}|{1}\rangle _{4}),\nonumber \\ |{00}\rangle _{12}&\longmapsto |{0}\rangle _{3}|{0}\rangle _{4}, \end{aligned}$$

where we assume the case of indistinguishable photons. For distinguishable photons the state $|{11}\rangle$ can also be represented by the transformations

51 $$\begin{aligned} |{11}\rangle _{12}&\longmapsto |{1}\rangle _{3}|{1}\rangle _{4} \end{aligned}$$

52 $$\begin{aligned} {|}{ {11}}\rangle _{12}&{\longmapsto \frac{1}{\sqrt{2}}(|}{ {0}}\rangle {_{3}|}{ {2}}\rangle {_{4}-|}{ {2}}\rangle _{3}|{{0}}\rangle _{4}). \end{aligned}$$

The Bell state measurement is considered successful when exactly one of the two detectors is triggered in each OAM mode. By taking into account the effects of detector dark counts $p_d$, we obtain the photon detection probabilities by conditioning on the following events;

*Dark counts*

In a case where no photons reach the input ports of the beam splitter, detection events can only result from detector noise. In this case, the detection probability is given by

53 $$\begin{aligned} {P_{\text {det}}(|00\rangle )=(1-\eta _{a})(1-\eta _{b})p_d^{2}(1-p_d)^{2}.} \end{aligned}$$

*One-photon case* Consider a case where only one photon form the two parties reach the input port of the beam splitter, then detection probability is given by

54 $$\begin{aligned} {P_{\text {det}}(|01\rangle )}&{=(1-\eta _{a})\eta _{b}(1-p_d)p_d(1-p_d)^{2}} \end{aligned}$$

55 $$\begin{aligned} {P_{\text {det}}(|01\rangle )}&{=\eta _{a}(1-\eta _{b})(1-p_d)p_d(1-p_d)^{2}} \end{aligned}$$

*Two-photon case* We now determine detection events emanating from two photons entering the beam splitter. The two photons can leave the BS at different ports or they may exit from the same port, and the detection probabilities are respectively given by

56 $$\begin{aligned} {P_{\text {det}}(|11\rangle )}&\, {=\eta _{a}\eta _{b}(1-p_d)^{2}(1-p_d)^{2}}\end{aligned}$$

57 $$\begin{aligned} {P_{\text {det}}(|11\rangle )}&\, {=\eta _{a}\eta _{b}(1-p_d)p_d(1-p_d)^{2}.} \end{aligned}$$

Thus, the yield $Y_{11}^{\textrm{L0R1}}$ is given by

58 $$\begin{aligned} Y_{11}^{\textrm{L0R1}}&= (1-p_d)^{2}[\eta _{a}\eta _{b}+(\eta _{b}+ \eta _{a}-4\eta _{a}\eta _{b})p_d+(1-2\eta _{a}-2\eta _{b}+4\eta _{a}\eta _{b})p_{d}^{2}]. \end{aligned}$$

An error is obtained in the cases where $\textrm{L0}$ and $\textrm{R0}$ or $\textrm{L1}$ and $\textrm{R1}$ click. The detection probabilities for these events is given by

59 $$\begin{aligned} {Y_{11}^{\textrm{L0R0}(\textrm{L1R1})}=}&{(1-p_d)^{2}[(\eta _{b} +\eta _{a}-\eta _{a}\eta _{b})p_d+(1-2\eta _{a}-2\eta _{b}+2\eta _{a}\eta _{b})p_{d}^{2}] }\end{aligned}$$

Thus, an error rate $e_{11}^{\textbf{S}}$ is given by

60 $$\begin{aligned} e_{11}^{\textbf{S}}Y_{11}&= Y_{11}^{\textrm{L0R0}}+Y_{11}^{\textrm{L1R1}}\nonumber \\&= 2(1-p_d)^{2}[(\eta _{a}+\eta _{b}-\eta _{a}\eta _{b})p_d+(1-2\eta _{a}-2\eta _{b}+2\eta _{a}\eta _{b})p_{d}^{2}]\nonumber \\&= e_0(1-p_d)^{2}[(\eta _{a}+\eta _{b}-\eta _{a}\eta _{b})p_d+(1-2\eta _{a}-2\eta _{b}+2\eta _{a}\eta _{b})p_{d}^{2}] \end{aligned}$$

where $e_0=\frac{1}{2}$ corresponds to the error rate of random erroneous detection. The overall gain $Q_{\mu }^{\textbf{V}}$ and the QBER, $E_{\mu }^{\textbf{V}}$ are evaluated in accordance with the method in Ref.^29^ with modifications as follows

61 $$\begin{aligned} Q_{\mu }^{\textbf{V}}&= [D_{\textrm{L0}}(1-D_{\textrm{L1}})+ (1-D_{\textrm{L0}})D_{\textrm{L1}}] [D_{\textrm{R1}}(1-D_{\textrm{R0}})+ (1-D_{\textrm{R1}})D_{\textrm{R0}}]+[D_{\textrm{L0}}D_{\textrm{L1}} (1-D_{\textrm{R0}})(1-D_{\textrm{R1}})]\nonumber \\&\quad +\, [D_{\textrm{R0}}D_{\textrm{R1}}(1-D_{\textrm{L0}})(1-D_{\textrm{L1}})]. \end{aligned}$$

Here, the detection probabilities for the four detectors are given by

62 $$\begin{aligned} D_{\textrm{L0}(\textrm{R0})}&=1-(1-p_d)\exp \Bigg (-\Bigg |e^{i\theta _{a}} \frac{\sqrt{\eta _{a}\mu _{a}}}{2}+e^{i\theta _{b}}\frac{\sqrt{\eta _{b}\mu _{b}}}{2}\Bigg |\Bigg ), \end{aligned}$$

63 $$\begin{aligned} D_{\textrm{L1}(\textrm{R1})}&=1-(1-p_d)\exp \Bigg (-\Bigg | e^{i\theta _{a}}\frac{\sqrt{\eta _{a}\mu _{a}}}{2}-e^{i\theta _{b}}\frac{\sqrt{\eta _{b}\mu _{b}}}{2}\Bigg |\Bigg ). \end{aligned}$$

We adopt the following notation to simplify our analysis;

$$\begin{aligned}{}&\lambda =\eta _{a}\mu _{a}+\eta _{b}\mu _{b},\\&\Delta \theta =\theta _{b}-\theta _{a},\\&x=\sqrt{\eta _{a}\mu _{a}\eta _{b}\mu _{b}}/2,\\&y=(1-p_d)e^{-\lambda /4}, \end{aligned}$$

where $\lambda$ denotes the average number of photons after interference in the BS, and $\Delta \theta$ corresponds to the difference between Alice’s and Bob’s random overall phases. As a result, the probability of detection simplifies as follows

64 $$\begin{aligned} D_{\textrm{L0}(\textrm{R0})}&=1-ye^{-x\cos \Delta \theta }, \end{aligned}$$

65 $$\begin{aligned} D_{\textrm{L1}(\textrm{R1})}&=1-ye^{x\cos \Delta \theta }. \end{aligned}$$

The QBER, $E_{\mu }^{{\textbf{V}}}$ is expressed as

66 $$\begin{aligned} E_{\mu }^{{\textbf{V}}}Q_{\mu }^{{\textbf{V}}}=2D_{{\textrm{L0}}} (1-D_{{\textrm{L1}}})(1-D_{\textrm{R1}})D_{{\textrm{R0}}}. \end{aligned}$$

## Data Availability

The datasets generated during and/or analysed during the current study are available from the corresponding author on reasonable request.
